# CD271^+^ Human Mesenchymal Stem Cells Show Antiarrhythmic Effects in a Novel Murine Infarction Model

**DOI:** 10.3390/cells8121474

**Published:** 2019-11-20

**Authors:** Haval Sadraddin, Ralf Gaebel, Anna Skorska, Cornelia Aquilina Lux, Sarah Sasse, Beschan Ahmad, Praveen Vasudevan, Gustav Steinhoff, Robert David

**Affiliations:** 1 Department of Cardiac Surgery, Rostock University Medical Center, 18059 Rostock, Germany; haval.sadraddin@evkln.de (H.S.); anna.skorska@med.uni-rostock.de (A.S.); cornelia.lux@gmx.de (C.A.L.); sarah-sasse@t-online.de (S.S.); beschan.ahmad@med.uni-goettingen.de (B.A.); praveen.vasudevan@med.uni-rostock.de (P.V.); gustav.steinhoff@med.uni-rostock.de (G.S.); robert.david@med.uni-rostock.de (R.D.); 2 Department Life, Light & Matter (LL&M), University of Rostock, 18057 Rostock, Germany

**Keywords:** arrhythmia, electrocardiography, cardiac regeneration, stem cells

## Abstract

Background: Ventricular arrhythmias (VA) are a common cause of sudden death after myocardial infarction (MI). Therefore, developing new therapeutic methods for the prevention and treatment of VA is of prime importance. Methods: Human bone marrow derived CD271^+^ mesenchymal stem cells (MSC) were tested for their antiarrhythmic effect. This was done through the development of a novel mouse model using an immunocompromised Rag2^−/−^ γc^−/−^ mouse strain subjected to myocardial “infarction-reinfarction”. The mice underwent a first ischemia-reperfusion through the left anterior descending (LAD) artery closure for 45 min with a subsequent second permanent LAD ligation after seven days from the first infarct. Results: This mouse model induced various types of VA detected with continuous electrocardiogram (ECG) monitoring via implanted telemetry device. The immediate intramyocardial delivery of CD271^+^ MSC after the first MI significantly reduced VA induced after the second MI. Conclusions: In addition to the clinical relevance, more closely reflecting patients who suffer from severe ischemic heart disease and related arrhythmias, our new mouse model bearing reinfarction warrants the time required for stem cell engraftment and for the first time enables us to analyze and verify significant antiarrhythmic effects of human CD271^+^ stem cells in vivo.

## 1. Introduction

Deaths arising from ventricular arrhythmias (VA) after acute cardiac events, in Germany, have increased by 100% over the last twenty years [1]. Therefore, new therapeutic methods for the prevention of VA following myocardial infarction (MI) need to be developed. There are reports that have indicated that transplanting of various stem cell populations post MI can influence the heart rhythm. Some have been defined as pro-arrhythmogenic cells, i.e., skeletal myoblasts, while others have shown antiarrhythmic effects, such as embryonic cardiomyocytes and mesenchymal stem cells (MSC) [2,3,4,5]. Our research group has focused on the role of human bone marrow (BM) derived hematopoiesis such as MSC in regenerative medicine [6,7,8,9]. In previous studies we have shown that it is safe to use CD117^+^ stem cells for myocardial regeneration, as their electrophysiological properties make them very unlikely to produce hazardous action potentials or contribute to arrhythmias [10,11]. However, the availability of a realistic small animal model reflecting the situation in patients is of utmost importance. For testing the rhythmological behavior of human cells, Rag2^−/−^ γc^−/−^ has been selected. These mice (strain C, 129S4-*Rag2^tm1.1Flv^Il2rg^tm1.1Flv^*/J) have been derived from a V17 embryonic stem cell line (BALB/c × 129 heterozygote) targeted for the Rag2 and Il2rg genes and lack B, T, and natural killer (NK) cells [12]. They have a deletion of the common cytokine receptor γ chain (γc) gene, and thus reduced numbers of peripheral T and B lymphocytes, and absent natural killer cell (NK) activity. A genetic cross with a recombinase activating gene 2 (RAG2) deficient strain produced mice doubly homozygous for the γc and RAG2 null alleles (Rag2^−/−^ γc^−/−^) with a stable phenotype characterized by the absence of all T lymphocyte, B lymphocyte, and NK cell function [13]. This phenotype makes the completely a lymphoid strain useful for studies on human tissue xenotransplantation [14].

As previously shown, stem cell antiarrhythmic effects appear after completion of an approximately one-week engraftment period [4]. The major objective of the present study was to develop a mouse model that enables us to reproduce VA several days after an initial MI. In vivo electrophysiological mouse models are well established: Previously, Berul et al. established an open chest epicardial study of conduction properties with the ability to induce second and third degree heart blocks, but no induction of tachyarrhythmias after programmed stimulation [15]. Hagendorff et al. used a rapid transesophageal atrial stimulation with the ability to induce some types of heart block and atrial tachyarrhythmias with induction of VA [16]. Roell et al. used a method relying on burst and extra stimulus pacing mediated induction of VA, leading to induction of ventricular tachycardia (VT) in 38.9% of the non-infarcted control group and 96.4% of the infarcted group [4].

This mouse model can also be considered representative for patients that suffer from severe ischemic heart disease and related arrhythmias. In this respect the application of the second MI at various points in time will be of interest. 

## 2. Materials and Methods

### 2.1. Bone Marrow Aspiration

Informed donors gave written consent to the aspiration of their BM according to the Declaration of Helsinki. The ethical committee of the University of Rostock approved the presented study (registered as no. A201023) as of 29 April 2010. The BM samples were obtained by sternal aspiration from patients undergoing coronary artery bypass graft surgery at Rostock University, Germany. The donors had no BM or hematological diseases and were not receiving any immune suppressing medication. The amount of the aspirated BM ranged from 60–100 mL. The BM samples were received from four donors. Two donors got more than 200,000 isolated CD271^+^ MSC in their BM samples and each one has been used for 2 mice (100,000 cells/animal). Anticoagulation was achieved by heparinization with 250 i.E./mL sodium heparine (B. Braun Melsungen AG, Melsungen, Germany). 

### 2.2. Cell Isolation

The mononuclear cells (MNC) were isolated by density gradient centrifugation using Lymphocyte Separation Medium (LSM; 1.077g/L, PAA Laboratories GmbH, Pasching, Austria). The MNC were indirectly labeled with CD271 APC and anti-APC micro beads (Miltenyi Biotec, Bergisch Gladbach, Germany) and CD271^+^ cells were enriched by positive magnetic selection using the magnet activated cell sorting (MACS) system (Miltenyi Biotec).

### 2.3. Flow Cytometric Analysis

Purity and viability of all cell isolations were verified by flow cytometry. Antibodies were all mouse anti-human and appropriate mouse isotype antibodies were applied for validation of our gating strategy. Anti-CD271 allophycocyanine was obtained from Miltenyi Biotec. Anti-CD45 allophycocyanin-H7, anti-CD45 Horizon V500, anti-CD29 allophycocyanin, anti-CD44 peridinin-chlorophyll-protein Cyanine 5.5 tandem dye, anti-CD73 phycoerythrin, as well as 7-aminoactinomycin were from BD Biosciences (Heidelberg, Germany) and anti-CD105 Alexa Fluor 488 was purchased from AbD Serotec GmbH (Puchheim, Germany). Near-IR live dead stain and 4’,6-diamidino-2-phenylindole (DAPI) were obtained from Thermo Fisher Scientific (Waltham, Massachusetts, USA).

Cells were suspended in MACS buffer containing PBS, 2mM EDTA, and 0.5% bovine serum albumin. To reduce unspecific binding, FcR blocking reagent (Miltenyi Biotec) was added to all samples. Cells were incubated with the antibodies for 10 min in the dark at 4 °C. RBC lysis buffer (red blood cell, eBioscience GmbH, Frankfurt/Main, Germany) was added for 10 min on ice. Samples were analyzed by BD LSRII flow cytometer and data were analyzed using FACSDiva software, version 6.1.2 (both Becton Dickinson, Franklin Lakes, New Jersey, USA). For optimal multicolor setting and correction of the spectral overlap, single stained controls were utilized, and gating strategy was performed with matched isotype/fluorescence minus one control. The CD271^+^ cells with a low granularity (SSC^low^) were included for further flow cytometric analysis and positivity for mesenchymal markers such as CD29, CD44, CD73, CD105, and CD271 was evaluated based on viable CD45^−^ cells in an established 6-fold staining, as previously described [17]. After performing antibody staining, as described above, 15 µM DAPI was added; cells were incubated for 2 min and then immediately acquired. 

### 2.4. Animals

All animal procedures were performed according to the guidelines from Directive 2010/63/EU of the European Parliament on the protection of animals used for scientific purposes. The federal animal care committee of LALLF Mecklenburg-Vorpommern (Germany) approved the study protocol (approval number LALLF M-V/TSD/7221.3-1-020/14). The Rag2^−/−^γc^−/−^ mice (strain C, 129S4-*Rag2^tm1.1Flv^Il2rg^tm1.1Flv^*/J) were purchased from the Jackson Laboratory (USA).

### 2.5. Ambulatory ECG Monitoring and Ligation of the Left Anterior Descending Artery

For ambulatory electrocardiogram (ECG) monitoring, twelve-to-fourteen-week-old female Rag2^−/−^γc^−/−^ mice (*n* = 22) were implanted with a telemetric device (Data Sciences International, DSI, New Brighton, Minnesota, USA) 7 days before performing the myocardial intervention. The mice were anesthetized with pentobarbital (50 mg/kg, intraperitoneal). An incision was made on the left thorax along the ribs to insert a telemetric transmitter (TA11ETA-F10 Implant; DSI) into a subcutaneous pocket with paired wire electrodes placed over the thorax with leads tunneled to the right upper and left lower thorax. The heart rate, PR interval and QRS complex as the entities of an ECG were recorded using Ponemah Physiology Platform (DSI) until 48 h post infarction. Telemetric ECG signal was qualitatively analyzed, and VA were detected and counted with ECG auto 1.5.11.26 software (EMKA Technologies, Paris, France).

**Figure 1:**
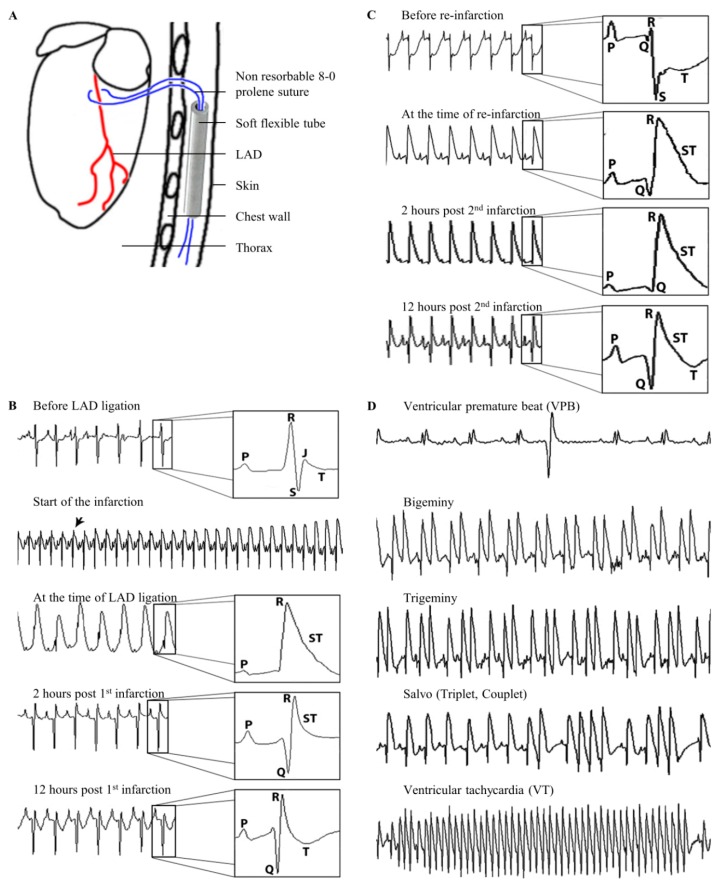
Induction of ventricular arrhythmias (VA). Schematic drawing of the loose left anterior descending (LAD) ligature positioning (**A**). Mouse ECG changes in relation to the time of the firs LAD ligation during ischemia-reperfusion infarction (**B**). Mouse ECG changes in relation to the time of the permanent second LAD-ligation (reinfarction and URI group **C**). Mouse ECG strips showing different types of observed VA (**D**).

Mice were randomly assigned into three groups: (untreated reinfarction, URI; stem cell treated reinfarction, SRI; and MI control, MIC). First, animals of all three groups underwent thoracotomy, as well as the ligation of the left anterior descending (LAD) artery. After 45 min, each mouse received an intramyocardial application of 20 µL BD Matrigel^TM^ Matrix (BD Biosciences, San Jose, CA, USA) and MACS Buffer in a ratio of 1/2. For stem cell treatment (SRI group) a total of 100,000 CD271^+^ MSC were injected at 4 sites along the border of the blanched myocardium (25,000 cells per injection point). Subsequently, the node was reopened (here, ischemia-reperfusion) leaving the ligature in the heart looped around the LAD with their ends kept inserted in a soft flexible tube placed subcutaneously (Figure 1A).

After an observation period of 7 days, every mouse underwent a second thoracotomy and, subsequently, animals of URI, as well as the SRI group, underwent permanent LAD ligation at the same site and using the suture from the first ligation (here, reinfarction). The rubber tube has facilitated finding of the suture material which was easily exempted from the surrounding tissue. MIC operated mice underwent an identical second surgical procedure without LAD ligation. 

### 2.6. Basic ECG Parameters

To evaluate the antiarrhythmic mechanism of the CD271^+^ MSC the basic ECG parameters of the mouse groups were measured at different time points. This was performed automatically with the use of ecgAUTO v3.3.0.28 software after manual definition of the start of P wave, the start of Q wave, R wave, end of S wave, and end of T wave. Then, the analysis was done for many subsequent beats at a specific time point. We measured the RR-interval, QRS duration, and QT-interval in milliseconds. The corrected QT-intervals (QTc) were measured using the formula QTc = QT/(RR/100)^½^, as previously described [18]. The basic ECG parameters were measured at the time points (1) 48 h after the first LAD ligation, (2) immediately before, and (3) 48 h after the second intervention.

### 2.7. Definitions of Various Ventricular Arrhythmias

The first temporary occlusion of the LAD resulted in a rapid onset of hyper acute T waves along with ST-segment elevation, as observed in the ECG record. Through reopening of the LAD 45 min post ligation, and subsequent myocardial reperfusion, ST-segment returned back to the isoelectric line and a development of Q waves could be observed. Figure 1B shows the recorded ECG before LAD occlusion, at the time of LAD occlusion, immediately after reopening of the LAD and reperfusion, as well as 12 h after the induction of the MI with clear Q waves and recognizable T waves.

Figure 1C shows the onset of the second infarction after permanently ligating the suture already placed at the time of the first ischemia one week earlier. The ECG immediately prior to performing reinfarction through the second LAD ligation shows deep S waves with deeply depressed ST-segment 7 days after the first operation (ischemia-reperfusion). The induction of the second MI with permanent ligation of the LAD led into an immediate ST-segment elevation in the ECG with subsequent development of deep Q waves. Two hours after reinfarction the ECG shows, a still elevated ST-segment reflecting the permanent LAD ligation. At 12 h post the second MI, Q waves developed with still elevated ST-segment with reappearance of T waves. 

Both, the first and second infarctions resulted in the development of different types of VA, namely ventricular premature beats (VPB), ventricular bigeminies and trigeminies (BG/TG), ventricular salvos, as well as ventricular tachycardia (VT) (Figure 1D).

We have analyzed, named, and defined the observed VA in this mouse model in accordance with a standard that fits the previously established guidelines for labeling and describing each type of VA [19] as follows: 

#### 2.7.1. Ventricular premature beats

We have defined VPB, as a ventricular electrical complex which varies in voltage (i.e., height) or duration (i.e., width) from the preceding non-VPB ventricular complex and occurs prematurely in relation to it. This means that VPB are not preceded by a P wave if they appear during the early cardiac cycle or they have shorter PR-intervals than those of non-VPB if they appear later in the cardiac cycle. 

#### 2.7.2. Bigeminies and trigeminies

A minimum sequence of VPB, normal sinus beat, and VPB repeated at least three times has been defined as bigeminy. The number of repetitions has been defined as three in our model. Additionally, we have defined a sequence of VPB, two normal sinus beats, and a consecutive VPB with a repetition of three times as trigeminy.

#### 2.7.3. Salvos

The observation of two or three consecutive VPB has been defined as salvo [19].

#### 2.7.4. Ventricular tachycardia

VT is defined as a sequence of four or more consecutive VPB [19,20]. This varies between three or more consecutive VPB to ten or more consecutive VPB [21,22]. A detailed description of the morphological and durational subtypes and classifications of VT has not been performed in these mouse models.

### 2.8. Organ Harvesting

Every mouse underwent euthanization, 48 h after the second intervention by cervical dislocation. Each heart was removed, embedded in O.C.T.^TM^ Compound (Sakura Finetek, Alphen aan den Rijn, Netherlands) and snap frozen in liquid nitrogen. For further histological examination of the infarction area the heart tissue was divided into four horizontal levels from the apex to the base and cut into 5 µm thick slices. 

### 2.9. Human Cells Detection

For detection of human cells on mouse heart cryosections, monoclonal anti-human nuclei primary antibody (Chemicon, Temecula, CA, USA) was used in combination with M.O.M.^TM^ Mouse Ig Blocking Reagent and M.O.M.^TM^ Protein Concentrate following the instructions of Vector^®^ M.O.M.^TM^ Immunodetection Kit (Linaris, Dossenheim, Germany). Donkey anti-mouse Alexa Fluor^®^ 594 served as conjugated secondary antibody followed by counterstaining with DAPI (both from Thermo Fisher Scientific).

### 2.10. Infarction Size and Leukocytes Infiltration Area Analysis

Randomly chosen histological heart sections of four horizontal infarct levels were stained with Fast Green FCF (Sigma-Aldrich, Saint Louis, Missouri, USA) and Sirius Red (Chroma Waldeck GmbH & Co. KG, Münster, Germany) assessing tissue localization and distribution of connective fibers. Two contiguous levels of the heart (*n* = 6 for each group) which represent the major infarction ratio were quantitatively estimated using computer aided image analysis (AxioVision LE Rel.4.5 software, Carl Zeiss AG, Oberkochen, Germany). To evaluate leukocytes infiltration area 48 h after the second ligation, the two contiguous levels of the heart which represent the major infarct ratio were stained with Hematoxylin (Merck, Darmstadt, Germany) and Eosin (Thermo Shandon Ltd., Runcorn, UK) and representative images were taken using computerized planimetry (AxioVision LE Rel.4.5 software). 

### 2.11. Statistical Analysis

Statistical analysis was performed using SigmaStat (Version 3.5, Systat Software, San Jose, CA, USA) and IBM SPSS Statistics for Windows (Version 22.0., IBM Corp., Armonk, NY, USA). The comparisons of two experimental groups were performed using Mann–Whitney *U* test. *p* values ≤ 0.05 were considered as statistically significant. 

## 3. Results

### 3.1. Immunophenotypic Analysis of CD271^+^ Mesenchymal Stem Cells

**Figure 2:**
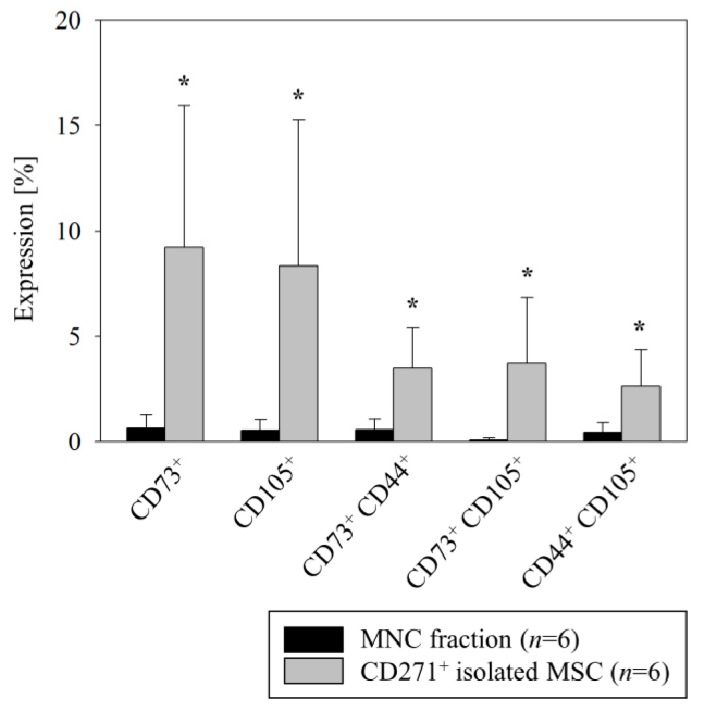
Flow cytometric analysis of MACS-isolated human BM CD271^+^ stem cells. The freshly isolated BM derived CD271^+^ stem cells showed a mesenchymal identity by a predominant expression of CD73 and CD105 MSC markers as compared with the entire MNC fraction. Mean ± SD, * *p* ≤ 0.015 (Mann–Whitney *U* Test).

We isolated CD271^+^ stem cells according to our established protocol which yields all mesenchymal colony forming progenitors [17,23]. Again, flow cytometric analysis confirmed a mesenchymal phenotype, reflected by significant overexpression of CD73 and CD105 markers in the isolated CD271^+^ cell fraction as compared with the entire BM-MNC fraction (9.2% ± 2.8% vs. 0.6% ± 0.2% for CD73 and 8.3% ± 2.8% vs. 0.5% ± 0.2% for CD105, respectively, Figure 2). Moreover, only a subfraction of CD271^+^ cells coexpressed all of the mesenchymal markers while being CD45^−^ (1.2% ± 0.3% CD271^+^CD44^+^CD73^+^CD105^+^ of CD271^+^ cells). 

### 3.2. Induced Ventricular Arrhythmias

Aiming to develop a murine in vivo model to study human stem cell transplantation, we found that VA are only detectable in the first day after induction of MI. To study engraftment and any alteration in the development of transplanted human cells in a murine model it is necessary to use an immunodeficient mouse strain. The trial of testing the potential antiarrhythmic effects of CD271^+^ MSC through catheter-based transjugular intracardial burst stimulation for induction of VA, a described method by Roell et al. [4], was not promising. We tested six immune compromised mice in which three mice underwent MI and three were healthy animals. All mice of the infarction group and the healthy group developed VA. This observation did not allow for the creation of a proper control group for comparison. Therefore, burst stimulation does not appear to be a suitable approach when using this mouse strain. Consequently, we developed a new mouse model in which we re-induce VA one week after a first ischemia reperfusion by performing a second permanent ligation. This served to simulate the situation in patients where the affected coronary vessel tends to reclose after successful initial recanalization.

In total, one animal (URI group) out of 22 mice died one-hour post induction of the second infarction, due to development of sinus, and, subsequently, third degree heart block. Consequently, this specimen was excluded from the statistical analysis. No other complications were observed after subcutaneous implantation of the telemeter and first LAD ligation, as well as the second LAD ligation.

### 3.3. Induction of Ventricular Arrhythmias after the Reinfarction

**Figure 3:**
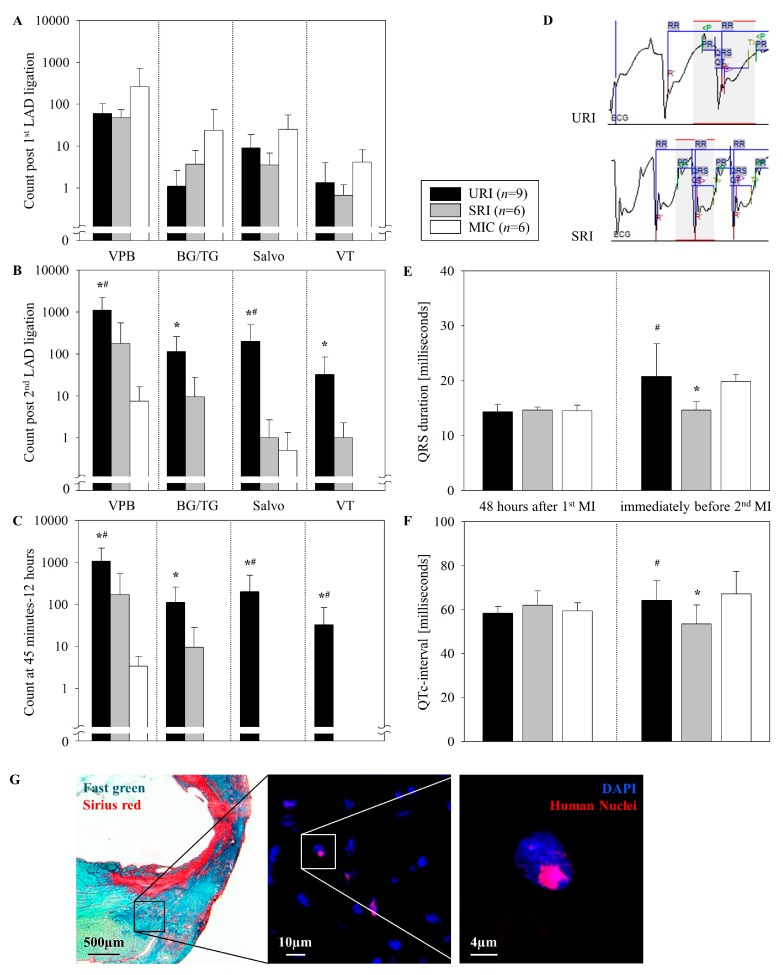
Comparison of developed VA. Until 12 h post LAD ligation (**A**,**B**). At the time period 45 min to 12 h post the second LAD ligation (**C**). ECG monitoring immediately prior to the second LAD ligation (**D**). QRS duration and QTc-interval 48 h post the first infarction and immediately prior to the second intervention (**E**,**F**). Mean ± SD, * *p* ≤ 0.05 as compared with MIC, ^#^ *p* ≤ 0.05 as compared with SRI (Mann–Whitney *U* Test). Representative images illustrate the engrafted human stem cells 9 days post transplantation performed for the SRI experimental group which the remaining human MSC were found predominantly in the peri-infarct area as with the Fast Green and Sirius Red staining method (the left picture) confirmed (**G**).

To simplify the study of the mechanisms underlying the developed VA, the ECG analyses were subdivided into an acute phase until 15 min and 15 to 45 min post intervention, as well as a delayed phase after 12 h (Table 1), as previously classified by [24]. There were no significant differences in any of the evolved arrhythmias between the groups after the first infarction. Likewise, quantitatively, there was no observed difference in the frequency of the development of VPB, ventricular salvos, and BG/TG 12 h after the first myocardial insult (Figure 3A).

The quantitative assessment of VPB events within 12 h following the second LAD ligation (URI) revealed a significant difference as compared with the control group MIC (1105.0 ± 1146.72 vs. 7.5 ± 8.98, respectively, Figure 3B). This significant difference is also evident in the time frame between 45 min and 12 h after the permanent infarction (URI 1082.2 ± 1127.77 vs. MIC 3.3 ± 2.42, Figure 3C). There was no significant difference in the occurrence of VPB after the first LAD ligation of both groups (URI 60.1 ± 42.19 vs. MIC 259.0 ± 457.69) for the first 12 h and at the time point between 45 min and 12 h (URI 54.5 ± 39.85 vs. MIC 246.8 ± 440.84). 

Interestingly, while a significant occurrence of VT after performing the second LAD ligation was identified for URI (32.6 ± 52.5), one week after the onset of the first ligation, none of that was observed in the MIC. This occurred during the first 12 h after the second infarction (Figure 3B) and also in the time frame between 45 min and 12 h after the second infarction (Figure 3C). There was no significant difference in terms of the development of VT after the first infarction (URI 1 ± 2.6 vs. MIC 1.6 ± 1.8). 

### 3.4. Antiarrhythmic Effects of CD271^+^ MSC Engraftment

The intramyocardial implantation of human CD271^+^ MSC did not lead to a significant difference in the number of VA early after the first MI (Figure 3A), but showed antiarrhythmic effects by significantly reducing the quantitatively measured VPB which occurred after a reinfarction during the time frame between LAD ligation until 12 h, thereafter (URI 1105.0 ± 1146.72 vs. SRI 178.16 ± 370.12, Figure 3B). Such significant antiarrhythmic behavior was also reflected in a reduction of the number of VT 45 min after the second LAD ligation until 12 h post reinfarction (URI 32.6 ± 52.51 vs. SRI 0 ± 0, Figure 3C). Moreover, there was no significant difference in BG/TG and salvos occurrence between the two groups. 

In order to further assess the mechanism of antiarrhythmic effect, ECG monitoring was used. Accordingly, we measured the mean QRS duration and the corrected QT-interval (Figure 3D). Immediately prior to the second LAD ligation (seven days after the first intervention), the stem cell treated animals had a significantly shorter QRS duration (URI 20.77 ± 1.98 vs. SRI 14.66 ± 0.61 and MIC 19.83 ± 0.54 milliseconds, Figure 3E). The significant shorter QTc-interval in SRI (URI 64.25 ± 3 vs. SRI 53.54 ± 3.52 and MIC 67.25 ± 4.14 milliseconds, Figure 3F) could be secondary to the shorter QRS duration as the QRS duration is an integral part of QT duration. There was no statistically significant difference in the mean QRS durations and QTc between URI and MIC immediately prior to the second intervention, as well as between any of the groups 48 h after the first LAD ligation. 

After nine days, the engrafted human cells were successfully detected on SRI mouse heart cryosections predominantly in the peri-infarct area by immunofluorescent staining (Figure 3G). 

### 3.5. Alterations of the Infarct Scar

**Figure 4:**
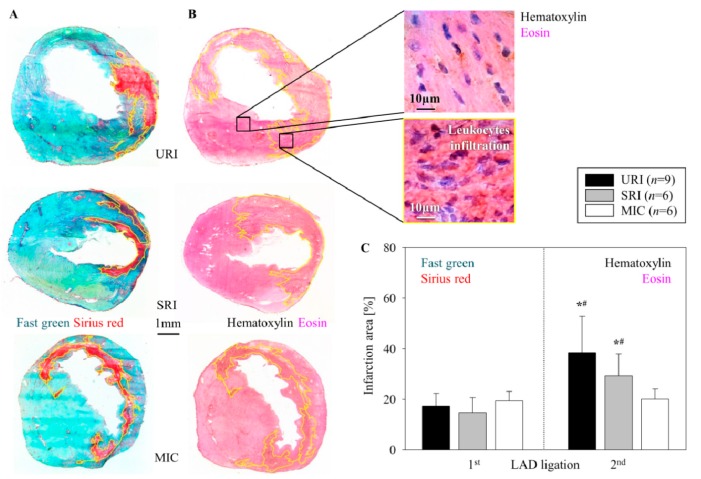
Alterations in MI size. Representative images show the infarction area (enclosed within the yellow border) for URI, SRI, and MIC 9 days after the first LAD ligation (Fast Green and Sirius Red staining) (**A**), as well as 48 h after the second LAD ligation (Hematoxylin and Eosin staining) (**B**). Significant increase of the infarction size after the second LAD ligation, mean ± SD, * *p* ≤ 0.009 as compared with the first LAD ligation, ^#^ *p* ≤ 0.041 in contrast to MIC (Mann–Whitney *U* test) (**C**).

The first ligation of the LAD and opening of the node after 45 min (ischemia-reperfusion) consistently resulted in a transmural MI with its typical histologic changes including the thinning of the left ventricular free wall (Fast Green) and extensive collagen deposition (Sirius Red) nine days post intervention (Figure 4A). After stem cell injection we observed no significant reduction in infarct scar formation (SRI, 14.78% ± 5.85%) in contrast to untreated infarction (URI 17.28% ± 5.10% URI), as well as to control group (MIC, 19.42% ± 3.66%, Figure 4C). 

The first ligation of the LAD followed by a permanent second LAD ligation resulted in leukocyte infiltration within the infarction area as depicted in the images of Hematoxylin and Eosin stained slices from hearts 48 h post infarction (Figure 4B). This cellular intervention consequently led to a significant enlargement of the myocardial scar area in URI (38.54% ± 14.28%), as well as SRI (29.36% ± 8.63%) in comparison to MIC (20.1% ± 4.04%, Figure 4C). 

## 4. Discussion

The purpose of this study was to develop a mouse model to reproduce VA that typically occurs in patients several days after an initial MI. To study engraftment and development of human cells in a murine model it was necessary to use an immunodeficient mouse strain. During development of an in vivo model of immunodeficient mice for the study of human stem cell transplantation, we found that VA are mostly seen only on the first day after induced MI. Our results supported the hypothesis, that a therapeutic stem cell treatment could have no such antiarrhythmic effect early after their transplantation, as previously shown for other cells [4]. For testing potential antiarrhythmic effects of intramyocardially transplanted cells, Roell et al. induced VA in vivo through transjugular venous catheter mediated burst stimulation of the myocardium after inducing MI [4]. However, this conventional approach could not be utilized in immunodeficient mice, because of the sensitivity of these animals as we observed. For this reason, there was an urgent need for the development of a new mouse model that used immunodeficient mice for in vivo study of antiarrhythmic effects exerted by various human-derived stem cells. On the basis of this, we aimed to develop a new mouse model with re-induced VA by performing a permanent second LAD ligation seven days after re-perfused of the first MI. Indeed, performing the second ligation, one week after the first infarction in Rag2^−/−^γc^−/−^ mice, reproduced cardiac arrhythmias. Other previously described small animal models in this field have utilized immune competent mice or rats subjected to higher stress after performing recurrent transient ischemia following an operatively instrumented mice with the capability of subsequent recurrent transient closure of the LAD [25,26]. 

The previously described development of VPB in the control group after anesthesia and surgery without performing LAD ligation [27] has also been seen in our mouse model in the time period between 15 and 45 min after re-thoracotomy (without the second LAD ligation) in MIC group. These developed VPB were statistically not significant as compared with the SRI and URI groups.

Our stem cell therapy resulted in shorter QRS duration, shorter QTc-intervals, and decreased occurrence of VT in the early period after the second MI. The observed shortening of QRS indicates the ability of MSC to improve cardiac electric conduction, which is in line with findings from Boink et al. who showed shortened QRS duration in a canine model of MI after MSC transplantation. [28]. However, antiarrhythmic behaviors of human MSC have also been shown in a clinical trial after their intravenous injection following a re-perfused MI [29]. The positive effect may be due to the possible coupling of the MSC with cardiomyocytes through connexin 43 (Cx43) bridges, as it has been found in an in vitro study by our research group [17]. This may be especially relevant as Cx43 plays a major role in post ischemia and post infarction cardiac arrhythmias in the time period between 45 min and 12 h after MI in different animal models [24]. 

As there is a close relationship between the infarct size and the incidence of VA [30], the significant reduction of the developed VA in the SRI group could not be attributed to the infarct size because there was no significant difference in the infarction area between the SRI and the URI group, early after the second infarction. The Hematoxylin and Eosin staining of the heart sections 48 h after the second intervention (second LAD ligation in SRI and URI groups, re-thoracotomy in MIC group) shows no infarct area expansion and new inflammatory cell infiltration in the MIC group. This indicates that the stained inflammatory cells in SRI and URI groups cannot be remaining cells of the initial MI and their infiltration is the early consequence of the second infarction. Additionally, the lack of intensive leukocyte infiltration in the MIC group after re-thoracotomy (no second LAD ligation) seen with Hematoxylin and Eosin staining of the heart sections nine days after the first operation (LAD ligation) in our mouse strain supports the finding of Yang et al. [31]. The described initial neutrophil infiltration in the infarct border one to two days after LAD ligation in immunocompetent C57BL/6J mice used by Yang and associates has been clearly seen in our SRI and URI groups 48 h after the second infarction. However, the described later lymphocytic infiltration, seven to 14 days after myocardial injury could not be noticeable in our complete alymphoid mouse strain, Rag2^−/−^γc^−/−^ nine days after induced MI.

In this study, we introduce our novel mouse model for testing arrhythmias in the Rag2^−/−^γc^−/−^ mouse. Importantly, CD271^+^ MSC transplanted into the infarcted area were retained and did not bear any proarrhythmic properties but rather antiarrhythmic effects. We have successfully utilized the model to test potential antiarrhythmic effects of human BM derived CD271^+^ stem cells in vivo and showed their safety and efficacy. Therefore, we conclude, that human BM derived MSC, i.e., CD271^+^ are suitable cell types to prevent arrhythmias after MI. This finding supports the previous observations on the safety of intramyocardial transplantation of BM derived stem cells and can be further evaluated for future clinical implication of cell transplantation in the field of electrophysiology [32]. Overall, our novel mouse model offers a new option for testing potential antiarrhythmic effects of cell transplantation therapies with high clinical relevance. 

## Figures and Tables

**Table 1:** Developed ventricular arrhythmias at various time points.

	First LAD Ligation (Mean ± SD)			Second LAD Ligation (Mean ± SD)		
	URI (*n* = 9)	SRI (*n* = 6)	MIC (*n* = 6)	URI (*n* = 9)	SRI (*n* = 6)	MIC (*n* = 6)
VPB						
0–12 h	60.1 ± 42.19	47.5 ± 25.8	259.0 ± 457.69	1105.0 ± 1146.72	178.16 ± 370.12	7.5 ± 8.98
0–15 min	1.8 ± 4.25	2.0 ± 3.63	7.5 ± 14.08	7.1 ± 7.97	4.0 ± 4.04	0.3 ± 0.81
15–45 min	3.6 ± 4.30	3.0 ± 4.69	4.6 ± 5.57	15.6 ± 22.75	0.33 ± 0.51	3.8 ± 7.22
45 min–12 h	54.5 ± 39.85	42.5 ± 27.38	246.8 ± 440.84	1082.2 ± 1127.77	173.8 ± 371.56	3.3 ± 2.42
BG/TG						
0–12 h	1.1 ± 1.45	3.66 ± 4.17	23.6 ± 49.35	113.8 ± 146.02	9.5 ± 18.41	0 ± 0
0–15 min	0 ± 0	0.16 ± 0.40	1.3 ± 3.26	0.5 ± 0.72	0 ± 0	0 ± 0
15–45 min	0.1 ± 0.33	0.16 ± 0.40	0.6 ± 1.21	0.6 ± 1.65	0 ± 0	0 ± 0
45 min–12 h	1.0 ± 1.50	3.33 ± 4.32	21.6 ± 45.89	112.6 ± 144.78	9.5 ± 18.41	0 ± 0
Salvos						
0–12 h	9.0 ± 9.89	3.5 ± 3.27	25.3 ± 29.96	201.7 ± 296.77	1.0 ± 1.67	0.5 ± 0.83
0–15 min	0 ± 0	1.16 ± 1.83	1.0 ± 1.54	0.3 ± 0.50	1.0 ± 1.67	0 ± 0
15–45 min	0.5 ± 0.88	0.5 ± 0.54	12.1 ± 0.64	0 ± 0	0 ± 0	0.5 ± 0.83
45 min–12 h	8.4 ± 10.17	1.83 ± 2.56	12.1 ± 18.17	201.4 ± 296.89	0 ± 0	0 ± 0
VT						
0–12 h	1.3 ± 2.69	0.66 ± 0.51	4.1 ±3.97	32.6 ± 52.51	1.0 ± 1.26	0 ± 0
0–15 min	0.5 ± 0.83	0.16 ± 0.40	0 ± 0	0 ± 0	0.83 ± 1.16	0 ± 0
15–45 min	0.3 ± 1.00	0.16 ± 0.40	2.0 ± 4.42	0 ± 0	0.16 ± 0.40	0 ± 0
45 min–12 h	1.0 ± 2.64	0.33 ± 0.51	1.6 ±1.86	32.6 ± 52.51	0 ± 0	0 ± 0

## References

[1] Herzstiftung eV D. 28 (2016). Deutscher Herzbericht. Deutsche Herzstiftung eV. www.herzstiftung.de/herzbericht.

[2] Smits P.C., van Geuns R.J.M., Poldermans D., Bountioukos M., Onderwater E.E.M., Lee C.H., Maat A.P.W.M., Serruys P.W. (2003). Catheter-based intramyocardial injection of autologous skeletal myoblasts as a primary treatment of ischemic heart failure: Clinical experiencewith six-month follow-up. J. Am. Coll. Cardiol..

[3] Hagège A.A., Marolleau J.P., Vilquin J.T., Alhéritière A., Peyrard S., Duboc D., Abergel E., Messas E., Mousseaux E., Schwartz K. (2006). Skeletal myoblast transplantation in ischemic heart failure: Long-term follow-up of the first phase I cohort of patients. Circulation.

[4] Roell W., Lewalter T., Sasse P., Tallini Y.N., Choi B.R., Breitbach M., Doran R., Becher U.M., Hwang S.-M., Bostani T. (2007). Engraftment of connexin 43-expressing cells prevents post-infarct arrhythmia. Nature.

[5] Hwang H.J., Chang W., Song B.-W., Song H., Cha M.J., Kim I.K., Lim S., Choi E.J., Ham O., Lee S.Y. (2012). Antiarrhythmic potential of mesenchymal stem cell is modulated by hypoxic environment. J. Am. Coll. Cardiol..

[6] Steinhoff G., Nesteruk J., Wolfien M., Kundt G., Börgermann J., David R., Garbade J., Große J., Haverich A., Hennig H. (2017). Cardiac function improvement and bone marrow response outcome analysis of the randomized perfect phase III clinical trial of intramyocardial CD133^+^ application after myocardial infarction. EBioMedicine.

[7] Mueller P., Gaebel R., Lemcke H., Wiekhorst F., Hausburg F., Lang C., Zarniko N., Westphal B., Steinhoff G., David R. (2017). Intramyocardial fate and effect of iron nanoparticles co-injected with MACS^®^ purified stem cell products. Biomaterials.

[8] Skorska A., Müller P., Gaebel R., Große J., Lemcke H., Lux C.A., Bastian M., Hausburg F., Zarniko N., Bubritzki S. (2017). GMP-conform on-site manufacturing of a CD133^+^ stem cell product for cardiovascular regeneration. Stem Cell Res. Ther..

[9] Gaebel R., Furlani D., Sorg H., Polchow B., Frank J., Bieback K., Wang W., Klopsch C., Ong L.-L., Li W. (2011). Cell origin of human mesenchymal stem cells determines a different healing performance in cardiac regeneration. PLoS ONE.

[10] Ludwig M., Skorska A., Tölk A., Hopp H.-H., Patejdl R., Li J., Steinhoff G., Noack T. (2013). Characterization of ion currents of murine CD117^+^ stem cells in vitro and their modulation under AT2R stimulation. Acta Physiol..

[11] Ludwig M., Tölk A., Skorska A., Maschmeier C., Gaebel R., Lux C.A., Steinhoff G., David R. (2015). Exploiting AT2R to improve CD117 stem cell function in vitro and in vivo-perspectives for cardiac stem cell therapy. Cell Physiol. Biochem..

[12] Song J., Willinger T., Rongvaux A., Eynon E.E., Stevens S., Manz M.G., Flavell R.A., Galán J.E. (2010). A mouse model for the human pathogen Salmonella typhi. Cell Host Microbe.

[13] Goldman J.P., Blundell M.P., Lopes L., Kinnon C., Di Santo J.P., Thrasher A.J. (1998). Enhanced human cell engraftment in mice deficient in RAG2 and the common cytokine receptor gamma chain. Br. J. Haematol..

[14] Mazurier F., Fontanellas A., Salesse S., Taine L., Landriau S., Moreau-Gaudry F., Reiffers J., Peault B., Di Santo J.P., De Verneuil H. (1999). A novel immunodeficient mouse model--RAG2 x common cytokine receptor gamma chain double mutants-requiring exogenous cytokine administration for human hematopoietic stem cell engraftment. J. Interf. Cytokine Res..

[15] Berul C.I., Aronovitz M.J., Wang P.J., Mendelsohn M.E. (1996). In vivo cardiacelectrophysiology studies in the mouse. Circulation.

[16] Hagendorff A., Schumacher B., Kirchhoff S., Lüderitz B., Willecke K. (1999). Conductiondisturbances and increased atrialvulnerability in connexin40-deficientmice analysed by transoesophageal stimulation. Circulation.

[17] Lemcke H., Gaebel R., Skorska A., Voronina N., Lux C.A., Petters J., Sasse S., Zarniko N., Steinhoff G., David R. (2017). Mechanisms of stem cell based cardiac repair-gap junctional signaling promotes the cardiac lineage specifcation of mesenchymal stem cells. Sci. Rep..

[18] Mitchell G.F., Jeron A., Koren G. (1998). Measurement of heart rate and Q-T interval in the con-scious mouse. Am. J. Physiol..

[19] Curtis M.J., Hancox J.C., Farkas A., Wainwright C.L., Stables C.L., Saint D.A., Clements-Jeweryg H., Lambiaseh P.D., Billmani G.E., Janse M.J. (2013). The Lambeth Conventions (II): Guidelines for the study of animal and human ventricular and supraVA. Pharmacol. Ther..

[20] Walker M.J.A., Curtis M.J., Hearse D.J., Campbell R.W.F., Janse M.J., Yellon D.M., Cobbe S.M., Coker S.J., Harness J.B., Harron D.W.G. (1988). The Lambeth Conventions: Guidelines for the study of arrhythmias in ischaemia, infarction, and reperfusion. Cardiovasc. Res..

[21] Zipes D.P., Camm A.J., Borggrefe M., Buxton A.E., Chaitman B., Fromer M. (2006). ACC/AHA/ESC 2006 guidelines for management of patients with VA and the prevention of sudden cardiac death–executive summary. Eur. Heart J..

[22] Fiedler V.B. (1983). Reduction of myocardial infarction and dysrhythmic activity by nafazatrom in the conscious rat. Eur. J. Pharmacol..

[23] Quirici N., Soligo D., Bossolasco P., Servida F., Lumini C., Deliliers G.L. (2002). Isolation of bone marrow mesenchymal stem cells by anti-nerve growth factor receptor antibodies. Exp. Hematol..

[24] Wit A.L., Peters N.S. (2012). The role of gap junctions in the arrhythmias of ischemia and infarction. Heart Rhythm.

[25] Lujan H.L., DiCarlo S.E. (2014). Reperfusion-induced sustained ventricular tachycardia, leading to ventricular fibrillation, in chronically instrumented, intact, conscious mice. Physiol. Rep..

[26] Leprán I., Koltai M., Siegmund W., Szekeres L. (1983). Coronary artery ligation, early arrhythmias, and determination of the ischemic area in conscious rats. J. Pharmacol. Methods.

[27] Betsuyaku T., Kanno S., Lerner D.L., Schuessler R.B., Saffitz J.E., Yamada K.A. (2004). Spontaneous and inducible ventricular arrhythmias after myocardial infarction in mice. Cardiovasc. Pathol..

[28] Boink G.J.J., Lu J., Driessen H.E., Duan L., Sosunov E.A., Anyukhovsky E.P., Shlapakova I.N., Lau D.H., Rosen T.S., Danilo P. (2012). Effect of skeletal muscle Na^+^ channel delivered via a cell platform on cardiac conduction and arrhythmia induction. Circ. Arrhyth. Electrophysiol..

[29] Hare J.M., Traverse J.H., Henry T.D., Dib N., Strumpf R.K., Schulman S.P., Gerstenblith G., De Maria A.N., Denktas A.E., Gammon R.S. (2009). A Randomized, Double-Blind, Placebo-Controlled, Dose-Escalation Study of Intravenous Adult Human Mesenchymal Stem Cells (Prochymal) After Acute Myocardial Infarction. J. Am. Coll. Cardiol..

[30] Bhar-Amato J., Davies W., Agarwal S. (2017). Ventricular Arrhythmia after Acute Myocardial Infarc-tion: ‘The Perfect Storm’. Arrhyth. Electrophysiol. Rev..

[31] Yang F., Liu Y.H., Yang X.P., Xu J., Kapke A., Carretero O.A. (2002). Myocardial Infarction and Cardiac Remodelling in Mice. Exp. Physiol..

[32] Beeres S.L., Zeppenfeld K., Bax J.J., Dibbets-Schneider P., Stokkel M.P., Fibbe W.E., van der Wall E.E., Atsma D.E., Schalij M.J. (2007). Electrophysiological and arrhythmogenic effects of intramyocardial bone marrow cell injection in patients with chronic ischemic heart disease. Heart Rhythm.

